# *Macrobiotus rebecchii* sp. nov.: A New Limno-Terrestrial and Hermaphroditic Tardigrade from Kyrgyzstan

**DOI:** 10.3390/ani12212906

**Published:** 2022-10-23

**Authors:** Daniel Stec

**Affiliations:** Institute of Systematics and Evolution of Animals, Polish Academy of Sciences, Sławkowska 17, 31-016 Kraków, Poland; daniel.stec@isez.pan.krakow.pl

**Keywords:** body granulation, hermaphrodite, integrative taxonomy, Kyrgyzstan, new species, tardigrades

## Abstract

**Simple Summary:**

In a moss sample collected on a rock in Kyrgyzstan, I discovered a new hermaphroditic tardigrade belonging to the genus *Macrobiotus* C.A.S. Schultze, 1834. To document this new species (*Macrobiotus rebecchii* sp. nov.) I used detailed morphological data collected from animals and eggs under a contrast phase light microscope (PCM) and scanning electron microscope (SEM). I also obtained DNA sequences from specimens of the new species (18S rRNA, COI). The external appearance of *Macrobiotus rebecchii* sp. nov. is similar to *Macrobiotus joannae* Pilato & Binda, 1983, *Macrobiotus punctillus* Pilato, Binda & Azzaro, 1990 and *Macrobiotus hannae* Nowak & Stec, 2018, but it can be easily differentiated from them mainly by a different body granulation pattern.

**Abstract:**

A new tardigrade species of the genus *Macrobiotus* C.A.S. Schultze, 1834 from Kyrgyzstan, is described and illustrated in this paper. *Macrobiotus rebecchii* sp. nov. is a hermaphroditic and limnoterrestrial species found in a moss growing on a rock in Toluk village. Specimens of the new species were examined for its morphological details using contrast phase light microscope (PCM) and scanning electron microscope (SEM). Genetic data in the form of DNA sequences of commonly used molecular markers were also obtained (18S rRNA, COI). Phenotypically the new species is most similar to *Macrobiotus joannae* Pilato & Binda, 1983, *Macrobiotus punctillus* Pilato, Binda & Azzaro, 1990, and *Macrobiotus hannae* Nowak & Stec, 2018, but can be easily differentiated from all of them by its body granulation pattern.

## 1. Introduction

The phylum Tardigrada includes aquatic micro-invertebrates represented by more than 1400 species [1,2,3]. Over a dozen new tardigrade species are described each year, expanding our knowledge, yet their biodiversity is poorly known. These animals have a global distribution and inhabit a wide variety of environments, from the greatest depths of the oceans to the highest mountain peaks, as well as extreme ephemeral habitats such as cryoconite holes and rock pools [4,5,6].

Although faunistic studies on the tardigrade fauna of Kyrgyzstan are rather scarce, several of them undertaken in the past two decades have made a considerable contribution to the taxonomy of Tardigrada. This is mainly due to the discovery of taxa new from this region, namely: *Isohypsibius borkini* Tumanov, 2003 [7], *Mesobiotus barabanovi* Tumanov, 2005 [8], *Macrobiotus kirghizicus* Tumanov, 2005 [8], *Milnesium asiaticum* Tumanov, 2006 [9] and *Milnesium reductum* Tumanov, 2006 [9], *Tenuibiotus danilovi* (Tumanov, 2007) [10], *Tenuibiotus tenuiformis* (Tumanov, 2007) [10], *Mesobiotus skorackii* Kaczmarek, Zawierucha, Buda, Stec, Gawlak, Michalczyk & Roszkowska, 2018 [11], *Macrobiotus caelestis* Coughlan, Michalczyk & Stec, 2019 [12], *Cornechiniscus* *imperfectus* Gąsiorek & Michalczyk 2020 [13], *Cornechiniscus mystacinus* Gąsiorek, 2022 [14], and a new genus being created with the discovery of *Cryoconicus kaczmareki* Zawierucha, Stec, Lachowska-Cierlik, Takeuchi, Z. Li & Michalczyk, 2018 [15]. Importantly, other studies have rediscovered and noted previously described species such as *Ramazzottius* cf. *oberhaeuseri*, which was reported by Vincente et al. [16], *M. reductum* reported by Morek et al. [17], *T. danilovi* and *T. tenuiformis* reported by Stec et al. [18], *M. kirghizicus* reported by Stec et al. [19], and three species of the genus *Cornechiniscus* Maucci & Ramazzotti, 1981 [20] (*C. cornutus* (Richters, 1907) [21], *C. lobatus* (Ramazzotti, 1943) [22], *C. subcornutus* Maucci & Ramazzotti, 1981 [20]) reported by Gąsiorek & Michalczyk [13].

In this article, I describe a new species of the genus *Macrobiotus*, *Macrobiotus rebecchii* sp. nov., discovered in a moss sample collected from a rock in Toluk village in Kyrgyzstan. The description is based on morphological and morphometric analyses conducted with the use of phase contrast light microscopy (PCM) and scanning electron microscopy (SEM). Phenotypic data of the new species are supplemented by DNA sequences of the two molecular markers (18S rRNA and COI) commonly used in tardigrade taxonomy.

## 2. Material and Methods

### 2.1. Sample Processing

A moss sample was collected from a rock in Toluk village in Kyrgyzstan in October 2018 by Barłomiej Surmacz and Witold Morek and was later examined for tardigrades using the protocol by Stec et al. [23]. To perform integrative taxonomic descriptions, the isolated animals and eggs extracted from the sample were split into three groups for specific analyses: morphological analysis with phase contrast light microscopy, morphological analysis with scanning electron microscopy, and DNA sequencing (for details please see section “Material examined” below).

### 2.2. Microscopy and Imaging

Specimens for light microscopy were mounted on microscope slides in a small drop of Hoyer’s medium and secured with a cover slip. Slides were kept at room temperature until the medium was dry and solid. The dried slides were sealed with transparent nail polish and examined under a Leica DMLB light microscope with PCM and a digital camera. Immediately after mounting the specimens in the medium, the slides were checked under PCM for the presence of males and females in the studied population, as the spermatozoa in the testis or ovotestis are visible only for a few hours after mounting [12,24]. To obtain clean and extended specimens for SEM, tardigrades were processed according to the protocol by Stec et al. [23]. Specimens were examined under high vacuum in a Versa 3D DualBeam SEM at the ATOMIN facility of the Jagiellonian University, Kraków, Poland. All figures were assembled in Corel Photo-Paint X6. For structures that could not be satisfactorily focused in a single light microscope photograph, a stack of two to six images with an equidistance of ca. 0.2 μm were manually assembled into a single deep-focus image in Corel Photo-Paint X6.

### 2.3. Morphometrics and Morphological Nomenclature

All measurements are given in micrometres (μm). The sample size was adjusted following the recommendations of Stec et al. [25]. Structures were measured only if their orientation was suitable. Body length was measured from the anterior extremity to the end of the body, excluding the hind legs. The terminology used to describe oral cavity armature and eggshell morphology follows Michalczyk & Kaczmarek [26] and Kaczmarek & Michalczyk [27]. The macroplacoid length sequence is given according to Kaczmarek et al. [28], while the morphological states of the cuticular bars on the legs follow Kiosya et al. [29]. The length of the buccal tube and the level of the insertion point of the stylet support were measured according to Pilato [30]. The *pt* index is the ratio of the length of a given structure to the length of the buccal tube expressed as a percentage [30]. All other measurements and nomenclature follow Kaczmarek & Michalczyk [27]. Specifically, the width of the buccal tube was measured as the external and internal diameter at the level of the stylet support insertion point. The heights of the claw branches were measured from the base of the claw (i.e., excluding the lunula) to the top of the branch, including accessory points. The distance between the egg processes was measured as the shortest line connecting the base edges of the two randomly chosen closest processes. Morphometric data were handled using the ‘Parachela’ ver. 1.8 template available from the Tardigrada Register [31] and are given in Supplementary Materials (Spreadsheets S1). The tardigrade taxonomy follows Bertolani et al. [32] and Stec et al. [18].

### 2.4. Genotyping

Before DNA isolation, two animals were temporarily mounted in a drop of water on microscope slide, secured with cover slip, checked under the light microscope, and immediately removed from the slide to avoid damage. DNA was extracted from the individual animals following a Chelex^®^ 100 resin (Bio-Rad) extraction method by Casquet et al. [33] with modifications described in detail by Stec et al. [34]. Two DNA fragments were sequenced: the small ribosome subunit (18S rRNA, nDNA), and the cytochrome oxidase subunit I (COI, mtDNA). All fragments were amplified and sequenced according to the protocols described by Stec et al. [34]. Primers and original references for specific PCR programs are listed in Table 1. The sequencing products were read with the ABI 3130xl sequencer at the Institute of Systematics and Evolution of Animals of the Polish Academy of Sciences, Kraków, Poland. Sequences were processed in BioEdit ver. 7.2.5 [35] and submitted to GenBank.

### 2.5. Morphological and Genetic Comparisons

For phenotypic comparison, the species most similar to the genus *Macrobiotus* were selected, namely: *Macrobiotus joannae* Pilato & Binda, 1983 [38] and *Macrobiotus hannae* Nowak & Stec, 2018 [39]. For genetic comparison, all published 18S rRNA and COI sequences of these taxa [32,39] were downloaded from GenBank. The sequences were aligned using the default settings (in the case of COI) and the Q-INS-I method (in the case of 18S rRNA) of MAFFT version 7 [40,41] and manually checked against non-conservative alignments in BioEdit. The aligned sequences were then trimmed to 758 (18S rRNA), and 657 (COI), bp. All COI sequences were translated into protein sequences in MEGA11 [42] to check against pseudogenes. The uncorrected pairwise distances were calculated using MEGA11 and are provided in the Supplementary Materials (Spreadsheets S2).

## 3. Results

### 3.1. Description of the New Species

#### 3.1.1. Taxonomic Account

**Phylum:** Tardigrada Doyère, 1840 [43]

**Class:** Eutardigrada Richters, 1926 [44]

**Order:** Parachela Schuster, Nelson, Grigarick and Christenberry, 1980 [45]

**Superfamily:** Macrobiotoidea Thulin, 1928 [46] (in Marley et al. [47])

**Family:** Macrobiotidae Thulin, 1928 [46]

**Genus:***Macrobiotus* C.A.S. Schultze, 1834 [48]

***Macrobiotus rebecchii*sp. nov**.

ZooBank: urn:lsid:zoobank.org:act:5914AE9E-F1FE-4516-BD2D-77B6C5CB08DB 

(Table 2 and Table 3; Figure 1, Figure 2, Figure 3, Figure 4, Figure 5, Figure 6, Figure 7, Figure 8, Figure 9 and Figure 10)

#### 3.1.2. Material Examined

Thirty-six animals, 65 eggs mounted on microscope slides in Hoyer’s medium, 10 animals and 11 eggs examined in SEM, and two animals processed for DNA sequencing.

#### 3.1.3. Type Locality

41°55′11.76″ N, 73°37′58.8″ E; 1509 m asl: Toluk, Kyrgyzstan, moss growing on rock in mountains, coll. Bartłomiej Surmacz and Witold Morek, October 2018.

#### 3.1.4. Etymology

The species is named after Lorena Rebecchi, a world-renowned tardigrade specialist from the University of Modena and Reggio Emilia (Modena, Italy). She intensively studied reproduction in Tardigrada, including the ovotestis maturation pattern in the hermaphroditic species *Macrobiotus joannae* which is similar to the new species described in this study.

#### 3.1.5. Type Depositories

Holotype: slide KG.001.01 with 11 paratypes and 24 paratypes (slides: KG.001.*, where the asterisk can be substituted by any of the following numbers: 02–03) and 65 eggs (slides: KG.001.*: 04–08) are deposited at the Institute of Systematics and Evolution of Animals, Polish Academy of Sciences, Sławkowska 17, 31-016, Kraków, Poland.

#### 3.1.6. Animals

**Figure 1:**
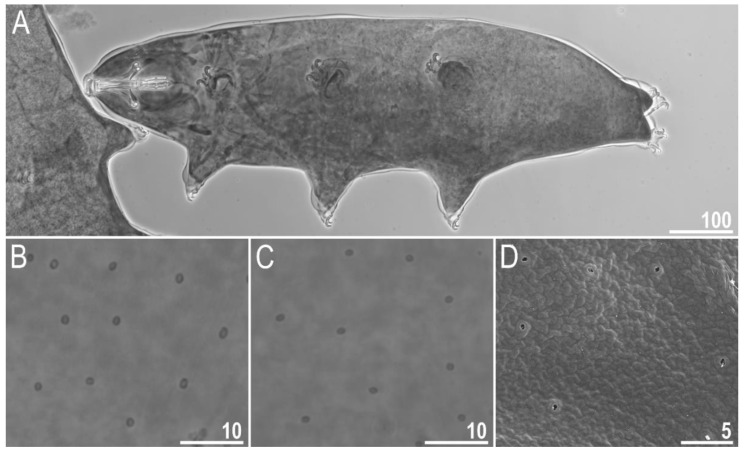
*Macrobiotus rebecchii* sp. nov.—images of habitus and cuticular pore: (**A**) dorso-ventral projection (holotype, PCM); (**B**) pores in the dorsal cuticle (paratype, PCM); (**C**) pores in the ventral cuticle (paratype, PCM); (**D**) pores in the dorsal cuticle (paratype, SEM). Panel D also shows minute granulation that is not visible in PCM (the same as in Figure 3F) but here it can hardly be discriminated from dirt, which is also present on this fragment of the dorsal cuticle. Scale bars in μm.

**Figure 2:**
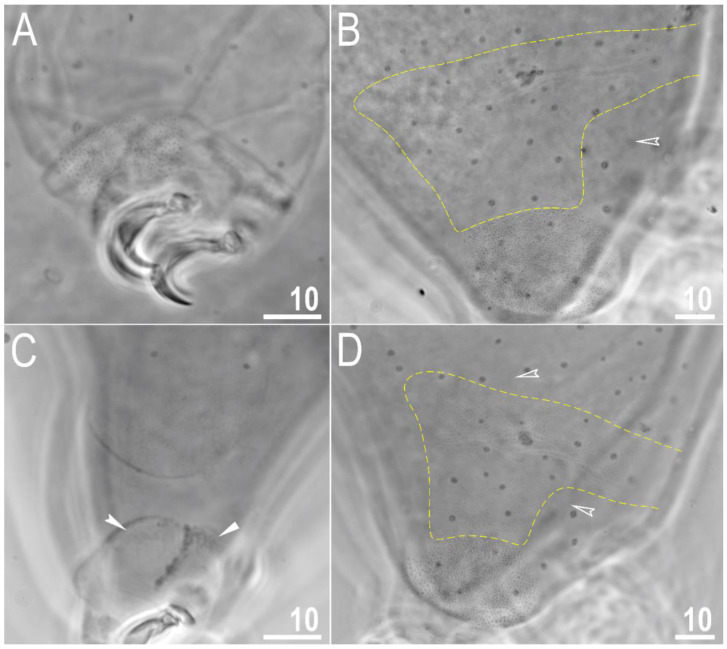
*Macrobiotus rebecchii* sp. nov. PCM images of leg and body granulation: (**A**) granulation on the external surface of leg III (paratype); (**B**) granulation on the hind leg and a band of sparse granulation seen in the caudal region of the last body segment (paratype); (**C**) a pulvinus-shaped cuticular bulge on the internal surface of leg II and an additional cuticular fold positioned distally (holotype); (**D**) granulation on the hind leg and a band of sparse granulation seen in the caudal region of the last body segment (paratype). The filled flat arrowhead indicates a single continuous cuticular bar above the claws, the filled indented arrowhead indicates an additional cuticular fold on the internal leg surface, and the empty indented arrowheads indicate body granulation faintly visible in PCM in the proximity of the well-visible caudal band of granulation, which is marked by a yellow dashed line. Scale bars in μm.

**Figure 3:**
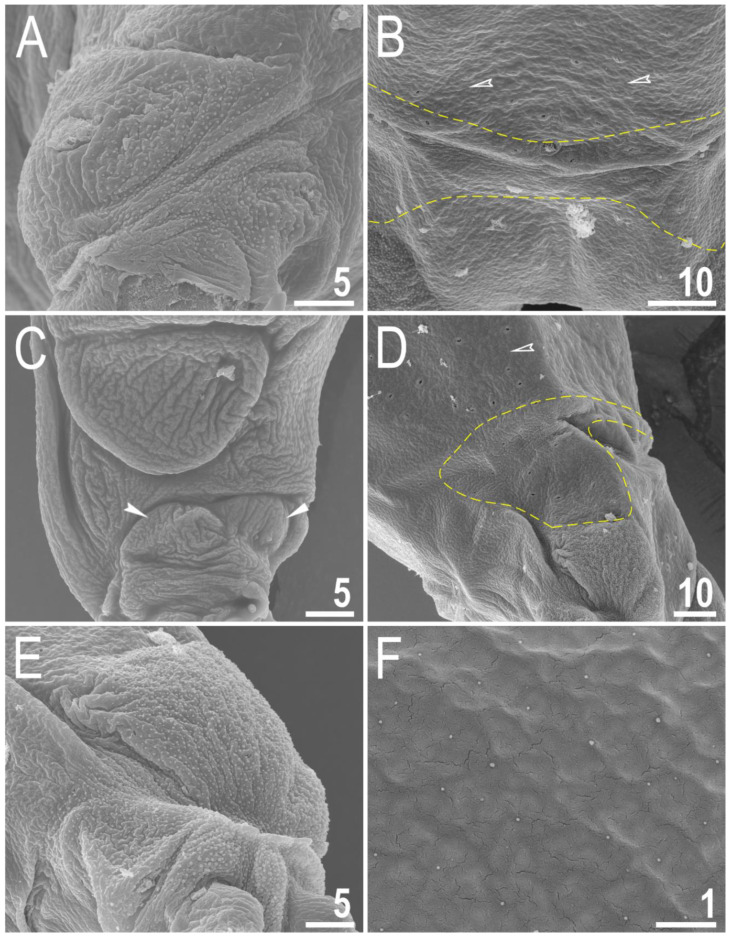
*Macrobiotus rebecchii* sp. nov. SEM images of leg and body granulation: (**A**) granulation on the external surface of leg III (paratype); (**B**) a band of sparse granulation seen in the caudal region of the last body segment (paratype); (**C**) a pulvinus-shaped cuticular bulge on the internal surface of leg I and an additional cuticular fold positioned distally (paratype); (**D**) granulation on hind leg and a band of sparse granulation seen in the caudal region of the last body segment (paratype); (**E**) granulation on hind leg (paratype); (**F**) body granulation on the dorsal cuticle that is not visible under PCM. The filled flat arrowhead indicates a single continuous cuticular bar above the claws, the filled indented arrowhead indicates an additional cuticular fold on the internal leg surface, and the empty indented arrowheads indicate body granulation that could potentially be faintly visible in PCM in the proximity of the well-visible caudal band of granulation, which is marked by a yellow dashed line. Scale bars in μm.

Body transparent in juveniles and white in adults, after fixation in Hoyer’s medium brownish (Figure 1A). Eyes present. Round and oval pores (0.4–0.7 μm in diameter), scattered randomly on the entire cuticle (on the ventral side of the body distributed more sparsely) (Figure 1B–D), including the external and internal surface of all legs. Evident granulation on the external surface of all legs I–III is visible under PCM and SEM (Figure 2A and Figure 3A). Granulation is also present on the lateral and dorsal surfaces of legs IV (Figure 2B,D and Figure 3D,E). A pulvinus-shaped cuticular bulge is present centrally on the internal surface of all legs I–III and an additional cuticular fold positioned distally (Figure 2C and Figure 3C). This structure is visible only if the legs are fully extended and well oriented on the slide or SEM stubs. In addition to the typical patches of leg granulation, a band of granulation is present on the dorso and latero-caudal surface of the last body segment (Figure 2B,D, Figure 3B,D and Figure 10C). This band of sparse dorsal granulation extends posteriorly and connects symmetrically with the granulation on both legs IV (Figure 2B,D, Figure 3B,D and Figure 10C). Leg granulation as well as caudal band of granulation are always clearly visible in PCM. However, except for this distinct granulation, the entire animal body is covered by evenly distributed, minute granulation, which is visible only under SEM (Figure 3F). The size of these microgranules, with diameters ranging from 0.05 to 0.07 μm, is below the resolution of the light microscope. This granulation can be slightly bigger only occasionally in the proximity of the mentioned well-visible caudal band, and on such occasions is very faintly visible in PCM (Figure 2B,D) but always well visible in SEM (Figure 3B,D).

**Figure 4:**
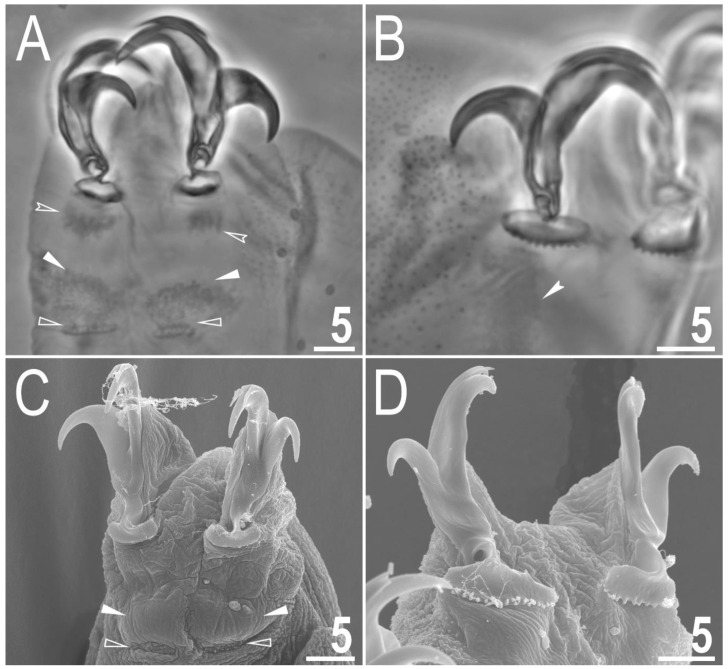
*Macrobiotus rebecchii* sp. nov. images of claws: (**A**) claws I with smooth lunulae (paratype, PCM); (**B**) claws IV with dentate lunulae (paratype, PCM); (**C**) claws II with smooth lunulae (paratype, SEM); (**D**) claws IV with dentate lunulae (paratype; SEM). The filled flat arrowheads indicate single continuous cuticular bars above the claws, the empty flat arrowheads indicate paired muscles attachments, the empty indented arrowheads indicate shadowed areas just above the lunulae, and the filled indented arrowhead indicates the horseshoe structure connecting the anterior and the posterior claw. Scale bars in μm.

Claws small and slender, of the *hufelandi* type (Figure 4A–D) with primary branches with distinct accessory points, a long common tract and an evident stalk connecting the claw to the lunula (Figure 4A–D). The lunulae in legs I–III are smooth (Figure 4A,C), while there is an evident dentation in the lunulae in legs IV (Figure 4B,D). A single continuous cuticular bar with central constriction is present above claws I–III (Figure 4A,C), with shadowed extensions narrowing toward double muscle attachments (Figure 4A; visible only in PCM). Sometimes, additional shadowed areas are present just above the lunulae (Figure 4A; visible only in PCM). A horseshoe-shaped structure connects the anterior and posterior lunules in leg IV (Figure 4B).

**Figure 5:**
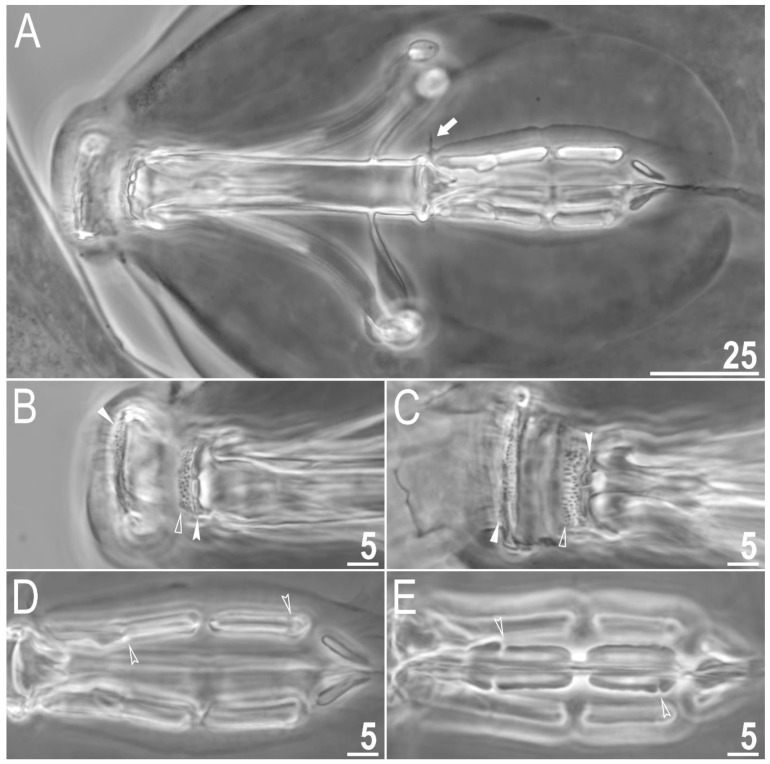
*Macrobiotus rebecchii* sp. nov. PCM images of the buccal apparatus: (**A**) an entire buccal apparatus (holotype); (**B**,**C**) the oral cavity armature, dorsal and ventral teeth respectively (paratypes); (**D**,**E**) placoid morphology, dorsal and ventral placoids respectively (holotype, paratype). The filled flat arrowheads indicate the first band of tenth, the empty flat arrowheads indicate the second band of teeth, the filled indented arrowheads indicate the third band of teeth, the empty indented arrowheads indicate central and subterminal constrictions in the first and second macroplacoid, respectively, and the arrow indicates anterior cuticular spike. Scale bars in μm.

**Figure 6:**
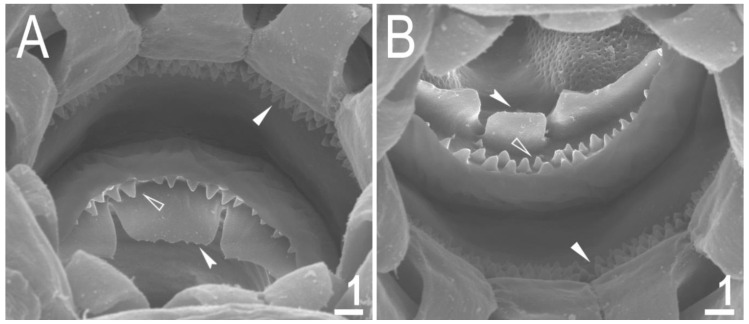
*Macrobiotus rebecchii* sp. nov. mouth opening and the oral cavity armature seen in SEM: (**A**,**B**) the oral cavity armature of a single paratype seen in SEM from different angles, dorsal (**A**) and ventral (**B**) view, respectively. The filled flat arrowheads indicate the first band of tenth, the empty flat arrowheads indicate the second band of teeth, and the filled indented arrowheads indicate the third band of teeth. Scale bars in μm.

Mouth antero-ventral. Bucco-pharyngeal apparatus of the *Macrobiotus* type, with the ventral lamina and ten small peribuccal lamellae followed by six buccal sensory lobes (Figure 5A and Figure 6A,B). Under PCM, the oral cavity armature is of the *hufelandi* type—three bands of teeth are always visible (Figure 5B,C). The first band of teeth is composed of numerous extremely small cones arranged in four to six rows located anteriorly in the oral cavity, just behind the bases of the peribuccal lamellae (Figure 5B,C and Figure 6A,B). The second band of teeth is located between the ring fold and the third band of teeth and comprises 4–5 rows of small cones, slightly larger than those of the first band (Figure 5B,C and Figure 6A,B). The teeth of the third band are located within the posterior portion of the oral cavity between the second band of teeth and the opening of the buccal tube (Figure 5B,C and Figure 6A,B). The third band of teeth is discontinuous and divided into the dorsal and ventral portions. Under PCM, the dorsal teeth are seen as three distinct transverse ridges, whereas the ventral teeth appear as two separate lateral transverse ridges between which a roundish median tooth is visible (Figure 5B,C). In SEM, both dorsal and ventral teeth are also clearly distinct (Figure 6A,B). Under SEM, the margins of the dorsal teeth are serrated (Figure 6A), whereas the margins of the ventral teeth are evidently less serrated (Figure 6B). Pharyngeal bulb spherical, with triangular apophyses, three anterior cuticular spikes (typically only two are visible in any given plane), two rod-shaped macroplacoids and a large triangular microplacoid (Figure 5A). The macroplacoid length sequence being 2 < 1. The first and the second macroplacoid are constricted centrally and subterminally, respectively (Figure 5D,E). Measurements and statistics are given in Table 2.

#### 3.1.7. Eggs

**Figure 7:**
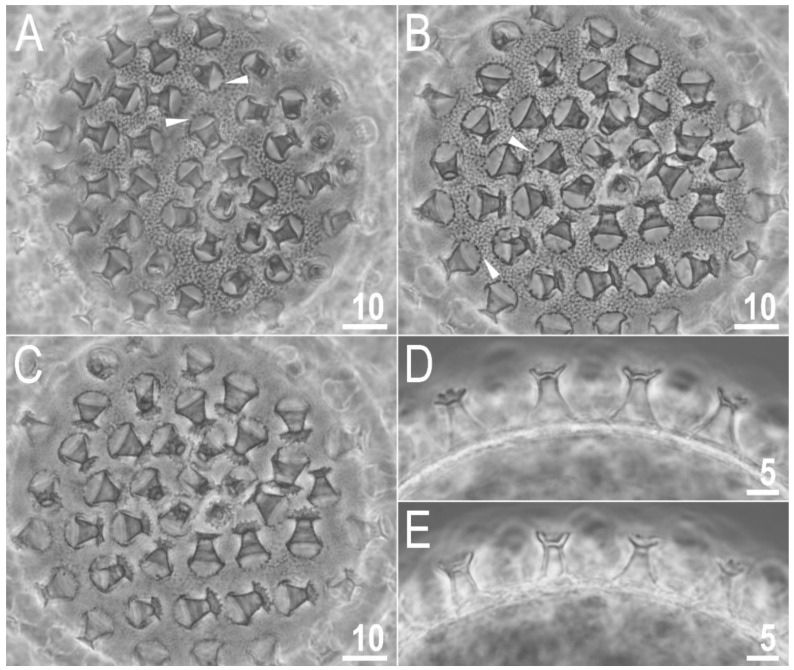
*Macrobiotus rebecchii* sp. nov. PCM images of the egg under ×1000 magnification. (**A**,**B**) egg surface; (**C**) egg surface, focus on egg processes and terminal discs; (**D**,**E**) midsections of egg processes. The filled flat arrowheads indicate thickenings around the processes bases. Scale bars in μm.

**Figure 8:**
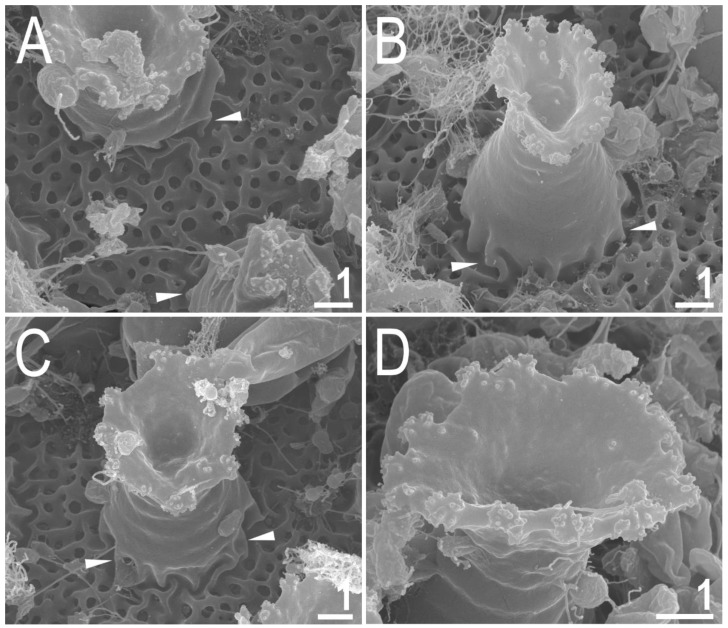
*Macrobiotus rebecchii* sp. nov. SEM images of eggs: (**A**–**C**) details of egg processes and the surface between them; (**D**) details of the terminal disc. The filled flat arrowheads indicate thickenings around the processes bases. Scale bars in μm.

Laid freely, white, spherical and ornamented (Figure 7A–E and Figure 8A–D). The surface between processes of the *hufelandi* type, i.e., chorion surface covered by evident reticulum (Figure 7A,B and Figure 8A–C). The reticulation is uniform across the entire surface. There are several rows of pores between processes, and the mesh nodes and bars are often wider than the pore diameter (the second character is more evident in SEM than in PCM; Figure 7A,B and Figure 8A–C). The pores in the reticulum are circular or slightly oval (0.25–0.60 μm in diameter), and under SEM almost all pores are seen to contain one or more small round or elongated granules (Figure 8A–C). The processes are in the shape of inverted goblets with slightly concave conical trunks and well-defined terminal discs (Figure 7A–E and Figure 8A–D). Faint annulations are visible under SEM on the process trunk (Figure 8A–D). A crown of gently marked thickenings is visible around the bases of the processes as darker dots in PCM (Figure 7A,B) and as wrinkled bases in SEM (Figure 7A–C). The terminal discs are cog-shaped, with a concave central area and 10–18 distinct teeth (Figure 7A–E and Figure 8A–D). The terminal discs, and especially their teeth, are covered by small granules (visible only under SEM) that probably serve to improve the adhesive properties of the egg processes (Figure 8A–D). Measurements and statistics are given in Table 3.

#### 3.1.8. Reproduction

**Figure 9:**
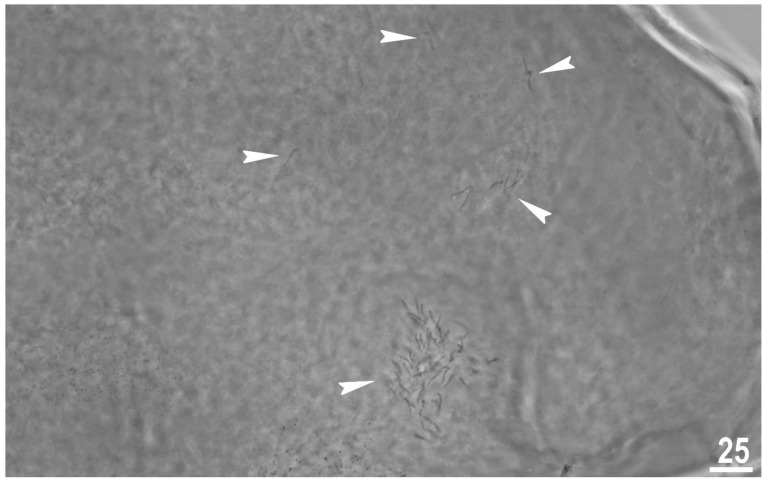
*Macrobiotus rebecchii* sp. nov. reproduction. Ovotestis filled with fully developed smermatozoa indicated by the filled indented arrowheads. Scale bar in μm.

The type population of *M. rebecchii* sp. nov. is hermaphroditic. In each of the analysed adult gravid individuals two types of gametes were observed. The observation of individuals freshly mounted in Hoyer’s medium revealed the ovotestis filled with spermatozoa (Figure 9) and developing oocytes.

#### 3.1.9. DNA Sequences and Comparison with Other Species

The 18S rRNA sequence (GenBank: OP479887, OP479888), 821 bp long;

The COI sequence (GenBank: OP477442, OP477443), 658 bp long.

The comparison of 18S rRNA sequences of the new species with sequences of *M. hannae* and *M. joannae* recovered no differences whereas COI sequences of the new species and *M. hannae* differ by 17%.

## 4. Discussion

**Figure 10:**
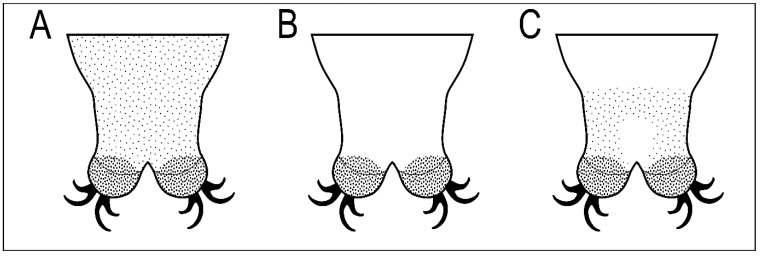
Schematic representation of three types of body granulation in the caudal region of the last body segment seen under light microscope (LM) in four *Macrobiotus* species: (**A**) *Macrobiotus joannae* Pilato & Binda, 1983 and *Macrobiotus punctillus* Pilato, Binda & Azzaro, 1990 (evenly distributed granulation clearly visible under LM); (**B**) *Macrobiotus hannae* Nowak & Stec, 2018 (body granulation granulation not visible under LM); (**C**) *Macrobiotus rebecchii* sp. nov. (granulation visible as a specific band on the dorso- and latero-caudal surface of the last body segment, while smaller granulation on the remaining body surface is not visible under LM).

The new species belongs to *Macrobiotus hufelandi* morphogroup sensu Stec et al. [18]. By having (i) three bands of teeth in the oral cavity armature that are well visible under light microscope, (ii) entire body cuticle covered by granulation (sometimes visible only in SEM), (iii) eggs with inverted goblet shaped processes and (iv) evident reticulation on the egg surface between processes, the new species is the most similar to three other *Macrobiotus* taxa, namely *M. joannae* reported from its type locality in Australia [38], and several uncertain localities in central, eastern, and south-eastern Russia [49] and from Italy [32], *M. hannae* known only from its type locality in Poland [39], and *Macrobiotus punctillus* Pilato, Binda & Azzaro, 1990 [50] known only from its type locality in Chile [50]. However, it can be easily distinguished from all of them by a different pattern of body granulation visible under a light microscope (LM; Figure 10). Specifically, *M. joannae* and *M. punctillus* have clearly visible granulation evenly covering the entire body surface (Figure 10A; [38,39,50]); *M. hannae* has minute body granulation evenly covering the entire body surface, but it is visible only in SEM and not in LM (Figure 10B; [39]), while *M. rebecchii* sp. nov. also has body granulation covering the entire body surface but comprises two types of granules: bigger granules that are visible under LM and form a specific band on the dorso- and latero-caudal surface of the last body segment, and smaller granulation which is not visible under LM (Figure 10C). The new species has also better developed and evident dentation in the lunulae of the hind legs compared to *M. hannae,* and very similar to those of *M. joannae* [39], whereas the lunulae of the hind legs are smooth in *M. punctillus* [50]. The new species also exhibits shadowed extensions of the cuticular bar above the claws in legs I–III narrowing toward double muscle attachments, which are absent in *M. hannae* and *M. joannae*. Although original descriptions of *M. joannae* and *M. hannae* did not report a crown of thickenings around the processes bases, they are visible in figures presented in Nowak & Stec [39]. Similarly, an additional cuticular fold positioned distally on the internal surface of legs I–III was not mentioned directly for *M. hannae* but is clearly visible in the figures provided in the original description [39]. In addition, there are no obvious differences in the morphometric characters between the new species and the *M. joannae* and *M. hannae*, as all its ranges and values do overlap. There are however small morphometric differences between *M. rebecchii* sp. nov**.** and *M. punctillus*, namely: larger body size (593–1061 µm in the new species vs. 285–397 µm in *M. puntillus*), larger eggs (86.0–110.1 µm and 101.0–125.6 µm for bare and full egg diameter in the new species vs. 70–71 µm and 83–84 µm for bare and full egg diameter in *M. punctillus*), higher and wider egg processes (6.5–9.3 µm and 5.2–8.4 µm for egg processes height and width in the new species vs. 5.0–5.5 µm and 4.9–5.2 µm for egg processes height and width in *M. punctillus*), and wider terminal discs on egg processes (4.8–8.2 µm in the new species vs. 3.7–3.9 µm in *M. punctillus*).

Genetic comparison and lack of differences between 18S rRNA sequences of the three compared species (*M. rebecchii* sp. nov., *M hannae*, *M. joannae*) confirmed their close relationship. This is also in agreement with their morphological similarity. Importantly, a high divergence in the mitochondrial marker between the new species and *M. hannae* demonstrates their distinctiveness and additionally supports the hypothesis of the new species. Such cases of extreme morphological similarity, lack of differences in nuclear markers, and evident interspecific divergence in COI, have been recently reported by several studies which described new macrobiotid taxa (e.g., [19,51,52,53,54,55,56]). Finally, the results presented herein, and the new species description from Kyrgyzstan, which is very similar to *M. joannae* and *M. hannae,* support the conclusions of Nowak & Stec [39] who questioned the validity of previous European records of *M. joannae*. Therefore, the confirmed geographic distribution of the three nominal hermaphroditic *Macrobiotus* species discussed in this study (*M. rebecchii* sp. nov., *M hannae*, *M. joannae*) seems to be limited to their type localities, unless other records are positively verified with the tools of integrative taxonomy.

## 5. Conclusions

The results of my study demonstrate morphological as well as genetic evidence for the distinctiveness of the newly found species from its congeners, supporting at the same time the erection of the new tardigrade species, *Macrobiotus rebecchii* sp. nov. As a result of this discovery, the number of tardigrade species recorded in Kyrgyzstan has now increased to 17. The new species is also the third *Macrobiotus* to be formally described in this country.

## Figures and Tables

**Table 1:** Primers with their original references used for amplification of the four DNA fragments sequenced in the study.

DNA Marker	Primer Name	Primer Direction	Primer Sequence (5′-3′)	Primer Source
18S rRNA	18S_Tar_Ff1	forward	AGGCGAAACCGCGAATGGCTC	[36]
	18S_Tar_Rr1	reverse	GCCGCAGGCTCCACTCCTGG	
COI	LCO1490-JJ	forward	CHACWAAYCATAAAGATATYGG	[37]
	HCO2198-JJ	reverse	AWACTTCVGGRTGVCCAAARAATCA	

**Table 2:** Measurements (in μm) and pt values of selected morphological structures of animals of *Macrobiotus rebecchii* sp. nov.; specimens mounted in Hoyer’s medium; N—number of specimen/structures measured. Range refers to the smallest and the largest structure among all measured specimens; SD—standard deviation.

CHARACTER	N	RANGE						MEAN		SD		Holotype	
		µm			*pt*			µm	*pt*	µm	*pt*	µm	*pt*
Body length	20	593	–	1061	*995*	*–*	*1489*	848	*1294*	108	*120*	902	*1348*
Buccal tube													
Buccal tube length	20	59.3	–	71.4		–		65.4	*–*	3.1	*–*	66.9	*–*
Stylet support insertion point	20	48.0	–	57.5	*79.0*	*–*	*81.6*	52.6	*80.5*	2.4	*0.6*	53.4	*79.8*
Buccal tube external width	20	10.4	–	13.1	*17.1*	*–*	*19.7*	12.1	*18.6*	0.7	*0.7*	12.4	*18.5*
Buccal tube internal width	20	8.4	–	10.9	*13.7*	*–*	*16.6*	9.7	*14.8*	0.7	*0.8*	10.1	*15.1*
Ventral lamina length	17	35.7	–	42.4	*54.4*	*–*	*66.4*	38.7	*59.1*	2.0	*3.0*	38.7	*57.8*
Placoid lengths													
Macroplacoid 1	20	19.9	–	27.5	*33.3*	*–*	*41.9*	24.0	*36.7*	2.1	*2.1*	25.6	*38.3*
Macroplacoid 2	20	12.6	–	16.1	*20.3*	*–*	*23.8*	14.4	*22.0*	1.1	*1.1*	15.7	*23.5*
Microplacoid	20	5.8	–	9.4	*9.0*	*–*	*14.7*	8.0	*12.2*	0.8	*1.1*	8.3	*12.4*
Macroplacoid row	20	34.3	–	55.1	*57.3*	*–*	*81.1*	40.9	*62.4*	4.4	*5.1*	43.4	*64.9*
Placoid row	20	43.1	–	53.9	*64.5*	*–*	*81.1*	49.8	*76.1*	3.8	*4.1*	53.5	*80.0*
Claw I heights													
External primary branch	20	14.1	–	19.4	*23.7*	*–*	*27.5*	17.1	*26.1*	1.3	*1.0*	17.8	*26.6*
External secondary branch	18	11.7	–	15.6	*18.6*	*–*	*22.0*	13.6	*20.8*	1.0	*0.9*	14.1	*21.1*
Internal primary branch	20	12.9	–	17.0	*21.6*	*–*	*25.2*	15.5	*23.7*	1.1	*1.1*	15.5	*23.2*
Internal secondary branch	17	10.3	–	13.2	*16.8*	*–*	*20.1*	12.1	*18.6*	0.7	*0.9*	12.8	*19.1*
Claw II heights													
External primary branch	20	14.2	–	19.6	*23.8*	*–*	*28.2*	17.5	*26.8*	1.3	*1.0*	18.6	*27.8*
External secondary branch	17	12.6	–	15.4	*19.7*	*–*	*23.0*	14.1	*21.4*	0.8	*0.9*	15.4	*23.0*
Internal primary branch	20	12.9	–	16.6	*21.6*	*–*	*25.6*	15.5	*23.7*	1.0	*1.0*	15.8	*23.6*
Internal secondary branch	19	10.5	–	14.3	*17.4*	*–*	*21.7*	12.8	*19.4*	0.9	*1.0*	13.3	*19.9*
Claw III heights													
External primary branch	20	13.9	–	20.2	*23.3*	*–*	*28.9*	17.7	*27.1*	1.3	*1.2*	17.8	*26.6*
External secondary branch	17	12.2	–	16.0	*20.6*	*–*	*23.0*	14.2	*21.7*	0.9	*0.7*	14.4	*21.5*
Internal primary branch	20	12.9	–	17.9	*21.6*	*–*	*25.9*	15.6	*23.9*	1.3	*1.1*	15.8	*23.6*
Internal secondary branch	19	10.9	–	14.9	*18.0*	*–*	*21.6*	12.8	*19.5*	1.0	*0.9*	13.0	*19.4*
Claw IV heights													
Anterior primary branch	18	15.5	–	20.0	*25.5*	*–*	*30.6*	18.2	*27.9*	1.2	*1.5*	18.0	*26.9*
Anterior secondary branch	15	12.3	–	16.4	*19.5*	*–*	*24.2*	13.8	*21.3*	1.0	*1.2*	13.6	*20.3*
Posterior primary branch	17	16.4	–	20.5	*26.1*	*–*	*31.3*	19.1	*29.4*	1.1	*1.4*	19.5	*29.1*
Posterior secondary branch	9	13.9	–	16.3	*21.1*	*–*	*24.2*	15.1	*22.9*	0.7	*1.1*	15.4	*23.0*

**Table 3:** Measurements (in μm) of the eggs of *Macrobiotus rebecchii* sp. nov.; eggs mounted in Hoyer’s medium; process base/height ratio is expressed as percentage; N—number of eggs/structures measured. Range refers to the smallest and the largest structure among all measured specimens; SD—standard deviation.

CHARACTER	N	RANGE			MEAN	SD
Egg bare diameter	20	86.0	–	110.1	97.9	5.3
Egg full diameter	20	101.0	–	125.6	114.3	5.6
Process height	60	6.5	–	9.3	8.1	0.8
Process base width	60	5.2	–	8.4	6.8	0.8
Process base/height ratio	60	67%	–	111%	84%	9%
Terminal disc width	60	4.8	–	8.2	6.3	0.6
Inter-process distance	60	2.8	–	6.9	4.6	0.9
Number of processes on the egg circumference	20	26	–	31	28.7	1.6

## Data Availability

The author confirms that the data supporting the findings of this study are available within the article and its supplementary materials. The DNA sequences generated in this study are available in GenBank.

## Supplementary Materials

The following supporting information can be downloaded at: https://www.mdpi.com/article/10.3390/ani12212906/s1. Spreadsheets S1. Raw morphometric data of *Macrobiotus rebecchii* sp. nov, Spreadsheets S2. P-genetic distances.
